# Design of a Glycoconjugate Vaccine Against *Salmonella* Paratyphi A

**DOI:** 10.3390/vaccines12111272

**Published:** 2024-11-12

**Authors:** Renzo Alfini, Martina Carducci, Luisa Massai, Daniele De Simone, Marco Mariti, Omar Rossi, Simona Rondini, Francesca Micoli, Carlo Giannelli

**Affiliations:** 1 GSK Vaccines Institute for Global Health (GVGH), 53100 Siena, Italy; martina.x.carducci@gsk.com (M.C.); luisa.x.massai@gsk.com (L.M.); daniele.x.desimone@gsk.com (D.D.S.); omar.x.rossi@gsk.com (O.R.); simona.x.rondini@gsk.com (S.R.); francesca.x.micoli@gsk.com (F.M.); carlo.x.giannelli@gsk.com (C.G.); 2 GSK Vaccines, 53100 Siena, Italy; marco.x.mariti@gsk.com

**Keywords:** *Salmonella* Paratyphi A, critical quality attribute (CQA), O-antigen (O:2), conjugate, 1-cyano-4-dimethylaminopyridine tetrafluoroborate (CDAP), immunogenicity, acetylation, molecular size

## Abstract

Background/Objectives: Typhoid and paratyphoid fever together are responsible for millions of cases and thousands of deaths per year, most of which occur in children in South and Southeast Asia. While typhoid conjugate vaccines (TCVs) are licensed, no vaccines are currently available against *S.* Paratyphi A. Here we describe the design of a *S.* Paratyphi A conjugate. Methods: The serovar-specific O-antigen (O:2) was linked to the CRM_197_ carrier protein (O:2–CRM_197_) and a panel of conjugates differing for structural characteristics were compared in mice and rabbits. Results: We identified the O-antigen molecular size, polysaccharide to protein ratio, conjugate cross-linking, and O:2 O-acetylation level as critical quality attributes and identified optimal design for a more immunogenic vaccine. Conclusions: This work guides the development of the O:2–CRM_197_ conjugate to be combined with TCV in a bivalent formulation against enteric fever.

## 1. Introduction

Infectious diseases continue to cause high morbidity and mortality throughout the world, and increasing rates of antimicrobial-resistant (AMR) bacteria cause high public health concerns. According to a recent report, 1.27 million deaths were directly attributed to AMR bacteria in 2019, with low- and middle-income countries being the most affected [1]. Vaccines can have a major role in fighting AMR bacteria: their use contributes not only to preventing infection but also limits the need for treatment and inappropriate use of antimicrobials, reducing the pressure for selecting resistant phenotypes and preserving the efficacy of antimicrobials [2,3,4]. Glycoconjugate vaccines represent a promising approach, as shown by several already licensed vaccines (*S*. Typhi, *S. pneumoniae*, *H. influenzae*, *N. meningitidis*, etc.) and the many more under development (*E. coli*, *S. aureus*, *K. pneumoniae*, *Shigella*, and *S.* Paratyphi A).

The vaccine against *S.* Typhi, based on the conjugation of the Vi capsular polysaccharide (PS) to tetanus toxoid, has shown very high efficacy [5] and has been recommended by the WHO in children from 9 months of age [6]. We have developed another typhoid conjugate vaccine (TCV), where Vi is conjugated to CRM_197_ carrier protein, which obtained WHO-prequalification in 2020 and is manufactured by Biological E (TyphiBEV). This vaccine has been introduced in routine immunization in Nepal and Malawi [7,8,9].

Although the majority of enteric fever cases are caused by *S.* Typhi, *S.* Paratyphi A remains an important contributing pathogen, and an increasing number of *S.* Paratyphi A infections have been observed across Asia. Estimates show that about a third of global enteric fever cases and >40% of cases in countries like India and Nepal are caused by *S.* Paratyphi A [10,11,12,13]. Antimicrobial-resistant *S.* Paratyphi A strains are on the rise, and the first case of *S.* Paratyphi A resistance to ceftriaxone has been reported in Pakistan [14].

To develop a vaccine against both typhoid and paratyphoid fever, we have generated a new glycoconjugate vaccine against *S.* Paratyphi A based on the serovar-specific O-antigen (O:2) conjugated to CRM_197_ carrier protein (O:2–CRM_197_) [15,16,17]. O:2 has been described as both an essential virulence factor and a protective antigen for *S.* Paratyphi A [16,17]; however, the OAg alone is not immunogenic but can induce anti-LPS antibodies with bactericidal activity after conjugation to a carrier protein [18].

The covalent linkage of the bacterial polysaccharide with an appropriate carrier protein results in glycoconjugate formation [19]. Different chemistries can be used, according to the polysaccharide-specific structures and characteristics [20,21]. Two main approaches are generally used: the polysaccharide is coupled to the protein through terminal group activation (site-selective approach), or the polysaccharide is activated randomly along the chain before linkage to the protein (random approach). For the TCV component, random chemistry was used to link the carboxylic groups of Vi polysaccharide to CRM_197_ derivatized with adipic di-hydrazide (ADH) by EDAC/NHS chemistry [9].

**Figure 1:**
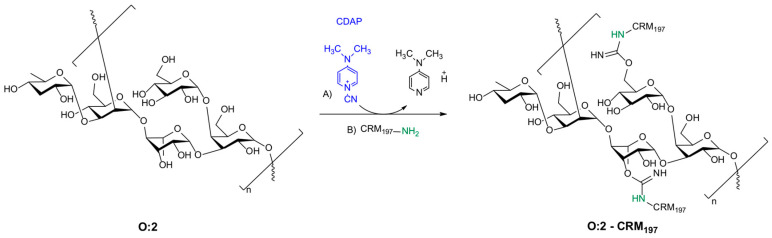
Chemical approach based on CDAP chemistry for the conjugation of O:2 to CRM_197_ carrier protein.

*S*. Paratyphi A O:2 consists of a trisaccharide backbone composed of rhamnose (Rha), mannose (Man), and galactose (Gal), with a branch of paratose (Par) from the C-3 of Man (conferring factor 2 serogroup specificity) and a branch of glucose (Glc) from the C-6 of Gal (Figure 1); C-3 of Rha is partially O-acetylated [22]. To conjugate O:2 to CRM_197_, we proposed a random approach with hydroxyl groups of O:2 randomly activated by 1-cyano-4-dimethylaminopyridine tetrafluoroborate (CDAP) before linkage to lysine residues of CRM_197_. The protein is directly added to the mixture without isolating the activated O:2 as an intermediate [23]. No linkers were used, avoiding additional derivatization steps of the carrier protein or the saccharide component (Figure 1). CRM_197_ (58.4 kDa) was selected as the carrier protein because of its well-established ability to provide T cell help, its safety profile, and the consistency of its large-scale manufacturability, which make it a very successful carrier for glycoconjugates [24].

The purpose of the current study was to identify O:2–CRM_197_ critical quality attributes, evaluating which parameters may affect its immunogenicity and stability. The results obtained by testing glycoconjugates varying in O:2 molecular size, O-acetylation level, O:2 activation degree, and O:2/CRM_197_ ratio in two different preclinical models are reported. The results allowed us to design a stable and immunogenic glycoconjugate against *S.* Paratyphi A.

## 2. Materials and Methods

### 2.1. Reagents

CRM_197_ (58.4 kDa) was obtained from GSK R&D (Siena, Italy).

### 2.2. O:2 Production, Purification, and Characterization

The O:2_[16kDa+100kDa]_ mixed population was produced from the ED199 ΔtolR strain as previously described [25]. The O:2_[16kDa]_ and O:2_[100kDa]_ were separated by SEC on a 1.6 × 90 cm S-300 HR column (Cytiva) eluting with PBS at 0.5 mL/min. Each population was collected and characterized by HPLC-SEC [15] for size determination; sugar content was quantified by HPAEC PAD [15] or by Dische colorimetric assay [26]. OAc content was measured by micro Hestrin assay, and % OAc was calculated as a molar ratio between nmol of OAc and nmol of Rha (Table 1).

### 2.3. Partial O:2 De-O-Acetylation Through Ammonia Treatment

Partial de-O-acetylation of O:2 was performed by treating the polysaccharide with a diluted solution of ammonia [27]. A solution of 1 mg/mL of O:2_[16kDa+100kDa]_ or O:2_[16kDa]_ in water was added with an ammonium hydroxide solution at a suitable concentration (NH_4_OH final concentration in the range of 5 ÷ 200 mM). The solution was kept at 25 °C for 1 h. Total O:2 de-O-acetylation was achieved after treatment with ammonia 1 M for 1 h at 37 °C. At the end of the reaction, an equal volume of Na_2_HPO_4_ (500 mM, pH 7.2) solution was added to quench the ammonium hydroxide. Partially de-O-acetylated O:2 and total de-O-acetylated O:2 were then buffer exchanged in the Amicon 3k device by three different washes against Na_2_HPO_4_ (200 mM, pH 7.2) and three subsequent washes against water.

### 2.4. Conjugation of S. Paratyphi A O:2 with CRM_197_

O:2 activation and conjugation were performed using CDAP with different O:2/CDAP/CRM_197_ ratios (Table 2) following the protocol described by Nappini et al. [23]. Measurement of the O:2 activation degree was performed using the protocol reported by Nappini et al. [23]. All conjugates were purified by hydrophobic interaction chromatography on a Phenyl HP column (Cytiva), loading the reaction mixture in 20 mM NaH_2_PO_4_/3 M NaCl (pH 7.2) [28]. The purified conjugate was eluted in water in 20 mM NaH_2_PO_4_ (pH 7.2), and the collected fractions were exchanged by ultrafiltration in the Amicon 10k in a suitable buffer (Tris 25 mM/NaCl 9 g/L, pH 7.2, or NaH_2_PO_4_ 20 mM/NaCl 100 mM, pH 7.2).

### 2.5. O:2–CRM_197_ Conjugates Characterization

#### 2.5.1. Conjugate MW Determination

Conjugates were analyzed by HPLC-SEC (80 μL injection) on a TOSOH TSKgel 5000 PW column using as eluent 0.1 M NaCl/0.1 M sodium phosphate (pH 7.2) containing 5% acetonitrile (ACN) at a flow rate of 0.5 mL/min. For MW determination, a refractive index detector was used with a dextran molecular weight standard (Sigma-Aldrich, St. Louis, MO, USA) calibration curve (50, 80, 150, 270, 410, and 670 kDa), performing a linear regression between log(MW) and retention time.

#### 2.5.2. *S*. Paratyphi A O-Antigen Quantification

OAg quantification was performed by measuring the Rha quantity with the Dische assay [26] (considering the polysaccharide repeating unit molecular weight) or by HPAEC PAD through the determination of the Rha content [29].

#### 2.5.3. Protein Quantification

Protein quantification was performed by microBCA assay. The amount of protein in each sample is determined by reading the 562 nm absorbance of the resulting product after mixing with reagents A, B, and C of the kit according to the manufacturer’s instructions (Pierce). Sample concentration is determined against a standard curve generated with bovine serum albumin in the range of 5–20 mg/mL, treated in the same conditions as the samples with linear fitting.

#### 2.5.4. Free CRM_197_ Determination in Conjugates

Conjugates were analyzed by HPLC-SEC (10 μL injection) on a TOSOH TSKgel 2000 SW-XL column using as eluent 0.1 M Na_2_SO_4_/0.1 M sodium phosphate (pH 6.6) at a flow rate of 0.3 mL/min. Unconjugated CRM_197_ was quantified using the fluorimeter detector (280 nm excitation/336 nm emission) with a calibration curve ranging from 10–100 μg/mL CRM_197_.

#### 2.5.5. Free Polysaccharide Quantification in Conjugates

A total of 1 mL of conjugate diluted to 100 μg/mL (in terms of protein) in 5 mM sodium phosphate (pH 7.2) with 150 mM NaCl and 0.005% Tween 20 was loaded on a C4 Solid Phase extraction disposable column (Vydac Bioselect C4). The solution was flowed by gravity or eventually by applying a positive air pressure over the liquid phase, and the flow through was collected. A total of 1 mL of 20% ACN with 0.05% TFA was loaded into the cartridge to maximize the free polysaccharide recovery, and the eluate was collected together with the initial flow through. The entire 2 mL volume collected was dried with a centrifugal evaporator and re-suspended in a suitable volume of water to quantify free saccharide by HPAEC PAD.

#### 2.5.6. Determination of the OAc Content of the O:2 and O:2–CRM_197_ Conjugates Through the Micro Hestrin Assay

The OAc content of the O:2 and O:2–CRM_197_ conjugates was determined by the Hestrin assay [30] carried out on a plate. O:2 and O:2–CRM_197_ were diluted to 0.5 mg/mL in terms of PS. Sample aliquots were also used to prepare blanks. A total of 114 μL of sample/standard/blanks were loaded by well. For both samples and blanks, the analysis was conducted in triplicate. A standard curve was prepared with acetylcholine (Sigma-Aldrich, St. Louis, MO, USA) in the range of 50–805 nmol/mL. In the case of O:2 sample wells and the calibration curve, 36 μL of basic hydroxylamine solution (hydroxylamine hydrochloride solution 4 M diluted 1.46× with NaOH 50%), 50 μL of HCl 4 M, and 50 μL of iron chloride 0.37 M (prepared in HCl 0.1 M) were added in the order indicated. For the sample blank wells, HCl 4 M was added before basic hydroxylamine. Bubbles on the plate were removed through a centrifugation step. In the case of O:2–CRM_197_, a further centrifugation step was added to remove the precipitate formed during the reaction. Supernatant post-centrifugation was then transferred onto the plate and read at 540 nm. O-Ac content was calculated as nmol/mL of OAc divided by the nmol/mL of O:2 repeating units (e.g., nmol/mL of Rha, measured by the Dische assay).

### 2.6. Immunogenicity Study in Animal Models

All animal sera used in this study were derived from mouse or rabbit immunization experiments performed at the Charles River Laboratories (France). The animal studies were reviewed by the local ethical committee (Project codes: 2017122815209931 v5 from 28 March 2018 (mice); 2016061011167092 from 11 September 2018 (rabbits)) and carried out in compliance with animal welfare standards according to European Directive 63/2010 and local legislation.

In the case of the mice studies, ten 5–6-week-old female CD-1 wild-type mice per group were injected intraperitoneally two times with a 200 μL/dose of 2.5 μg of O:2 at days 0 and 28 in the presence of Alhydrogel. In the case of the rabbit study, eight female (>1.5 kg) New Zealand White rabbits per group were injected intramuscularly with a 500 μL/dose of 25 μg of O:2 at days 0 and 28.

In all the animal studies, bleeds were collected at day 27 (post I), and the final bleed was collected at day 42 (post II). The collected blood was left at room temperature for decantation for 30 min, whereupon it was centrifuged at 2800 rpm for 15 min at +5 °C ± 3 °C.

Anti-O:2 IgG responses by ELISA SBA were performed according to procedures already published [31].

### 2.7. Formulation Preparation

For the first study in mice (investigation on the O:2 size, O:2/CRM_197_ *w*/*w* ratio, and cross-linking), the formulations were prepared by adding excipients and an antigen in the following order: water, Tris 100 mM (pH 7.4), saline 90 g/L, Alhydrogel (0.375 mg/mL final Al^3+^ concentration), and O:2–CRM_197_ conjugate. Each formulation was characterized by a final O:2 concentration of 12.5 μg/mL in Tris 10 mM (pH 7.4) and NaCl 9 g/L. Analysis of the supernatant of the formulations performed by SDS PAGE showed that in all formulations the quantity of conjugate not adsorbed on alum was lower than 10%.

Formulations with O:2–CRM_197_ conjugates at different OAc levels used to immunize mice were prepared with an O:2 concentration of 12.5 μg/mL in the presence of Alhydrogel (0.1875 mg/mL final Al^3+^ concentration), Tris 25 mM (pH 7.2), and NaCl 9 g/L. In this condition, O:2–CRM_197_ conjugates were adsorbed on Alhydrogel, with values of unabsorbed protein < 2.0% (by micro-BCA).

Formulations with O:2–CRM_197_ used to immunize rabbits were prepared similarly, but with a final O:2 and Alhydrogel concentration of 50.0 μg/mL and 0.75 mg/mL (in terms of Al^3+^), respectively. Also in this case, O:2–CRM_197_ conjugates were confirmed to be completely adsorbed on Alhydrogel (unabsorbed protein < 2.0% by micro-BCA).

### 2.8. Stability Test

The purified O:2–CRM_197_ conjugate obtained from O:2_[16kDa+100kDa]_ (with an initial O-acetylation content of 55.6%) was exchanged through Amicon 10k in two different buffers: Tris 20 mM/NaCl 9 g/mL (pH 7.2) or Histidine 10 mM (pH 6.5). The two resulting solutions were aliquoted in vials under a sterile hood. The different aliquots were put in stability at 5, 25, 32, and 40 °C. The aliquots were analyzed at days 0 (time 0 condition), 15, 45, 60, and 150 through the micro Hestrin and Dische assays to calculate the percentage of O-acetylation.

### 2.9. Statistical Analysis

Statistical analysis among/between groups was performed with GraphPad Prism 9.5.1 (GraphPad Software, Boston, MA, USA); a Mann–Whitney two-tailed test was used to compare the immune response elicited by two groups, and a Kruskal–Wallis test with Dunn’s post hoc analysis was used for comparison among more than two groups. The Wilcoxon matched-pairs signed rank two-tailed test was performed to compare the response induced by the same antigen at day 27 vs. day 42.

Regression analysis was performed using Minitab 18.1.0 (Minitab LLC, State College, PA, USA) or JMP 15.2.0 (SAS Institute Inc., Cary, NC, USA).

Kinetic modeling was performed using Thermokinetics 5.4 (AKTS), setting a 1-step model with the truncated Šesták–Berggren equation without accounting for eventual autocatalytic behavior. The model was optimized for the following parameters (Equation reported in Table 3): values of starting (T0) and final (T∞) O-acetylation, the pre-exponential factor (A), the activation energy (E), and the order of reaction (n). A prediction interval of 95% was calculated with the bootstrap method (n = 1000) resampling residual.

## 3. Results

### 3.1. Synthesis and Characterization of O:2–CRM_197_ Glycoconjugates

To evaluate the impact of O:2 size, O:2 activation degree, and O:2/CRM_197_ *w*/*w* ratio on immunogenicity, a panel of conjugates differing for these attributes was synthesized and fully characterized.

The *S*. Paratyphi A strain ED199 Δ*tolR* [32,33], used as a source of O:2, produces a bimodal molecular weight (MW) O-antigen population, with two main peaks at 16 kDa and 100 kDa, with a *w*/*w* ratio of 65:35 (Figure S1). The two populations, named O:2_[16kDa]_ and O:2_[100kDa]_, were separated by size exclusion chromatography (SEC), starting from the purified O:2_[16kDa+100kDa]_ mixed population. The two individual populations showed similar characteristics in terms of glucosylation and O-acetylation % (Table 1). The glucosylation level was not altered during SEC, while the O-acetylation level was partially impacted, decreasing from 60% in the O:2_[16kDa+100kDa]_ mixed population and from ~45% in O:2_[16kDa]_ and O:2_[100kDa]_ (Table 1).

Different O:2–CRM_197_ conjugates were synthesized using either O:2_[16kDa]_ (Conjugates 1–3, Table 2), O:2_[100kDa]_ (Conjugates 4–6, Table 2), or O:2_[16kDa+100kDa]_ (Conjugates 7–9, Table 2). Different O:2/CRM_197_/CDAP weight ratios were used during conjugation, resulting in a panel of conjugates differing not only in O:2 size but also in polysaccharide activation degree and O:2/CRM_197_ ratio (Table 2). After purification, all conjugates had a residual free O:2 below 5%.

Bacterial polysaccharides often contain O-acetyl esters (OAc), which may constitute an important part of the immunodominant epitopes. To evaluate the impact of O-acetylation on the immune response, we synthesized a panel of O:2–CRM_197_ conjugates with different O-acetylation levels (Table 4). Partial removal of the OAc groups from O:2 was performed before conjugation to CRM_197_ by treating O:2 with a weak base like ammonia [27]. The O:2_[16kDa+100kDa]_ mixed population was 60% O-acetylated. By treating the polysaccharide with increasing ammonia concentrations (range 5 ÷ 1000 mM) at 25 °C, we obtained a panel of partially or totally de-O-acetylated O:2, as shown in Table S1. By plotting the resulting O-acetylation level against the ammonia (NH_4_OH) concentration, a correlation function was established (Figures S2 and S3). Total O:2 de-O-acetylation was obtained using 1 M ammonia (Table S1).

Using the correlation function, O:2 was treated with suitable ammonia concentrations to obtain polysaccharides with the desired intermediate O-acetylation levels.

The starting O:2_[16kDa+100kDa]_ and the completely de-O-acetylated O:2_[16kDa+100kDa]_ were conjugated to CRM_197_, obtaining conjugates with 54% or 0% OAc, respectively (conjugates 1 and 5, Table 4). In addition, a selection of partially de-O-acetylated O:2 sugars were conjugated, resulting in a panel of O:2–CRM_197_ conjugates with 45.2%, 35.3%, and 18.5% OAc, respectively (conjugates 2-4, Table 4).

Similarly, O:2_[16kDa]_ and partially de-O-acetylated O:2_[16kDa]_ were conjugated to CRM_197_, resulting in two conjugates with 58.5% and 25.2% OAc, respectively (conjugates 6 and 7, Table 4).

O:2 activation by CDAP, followed by conjugation to CRM_197_, did not impact the initial O:2 OAc levels.

All conjugates were fully characterized and showed a similar O:2/CRM_197_ *w*/*w* ratio (Table 4) and level of cross-linking, as shown in the HPLC-SEC fluorescence emission profiles (Figure S4).

### 3.2. Immunogenicity of O:2–CRM_197_ Conjugates in Mice

The mice received two intraperitoneal (IP) immunizations, with a 28-day interval, of 2.5 μg of conjugates (O:2 dose). Anti-O:2 IgG response was determined by ELISA, and sera functional activity was determined by their ability to kill *S.* Paratyphi A bacteria in the presence of a complement.

**Figure 2:**
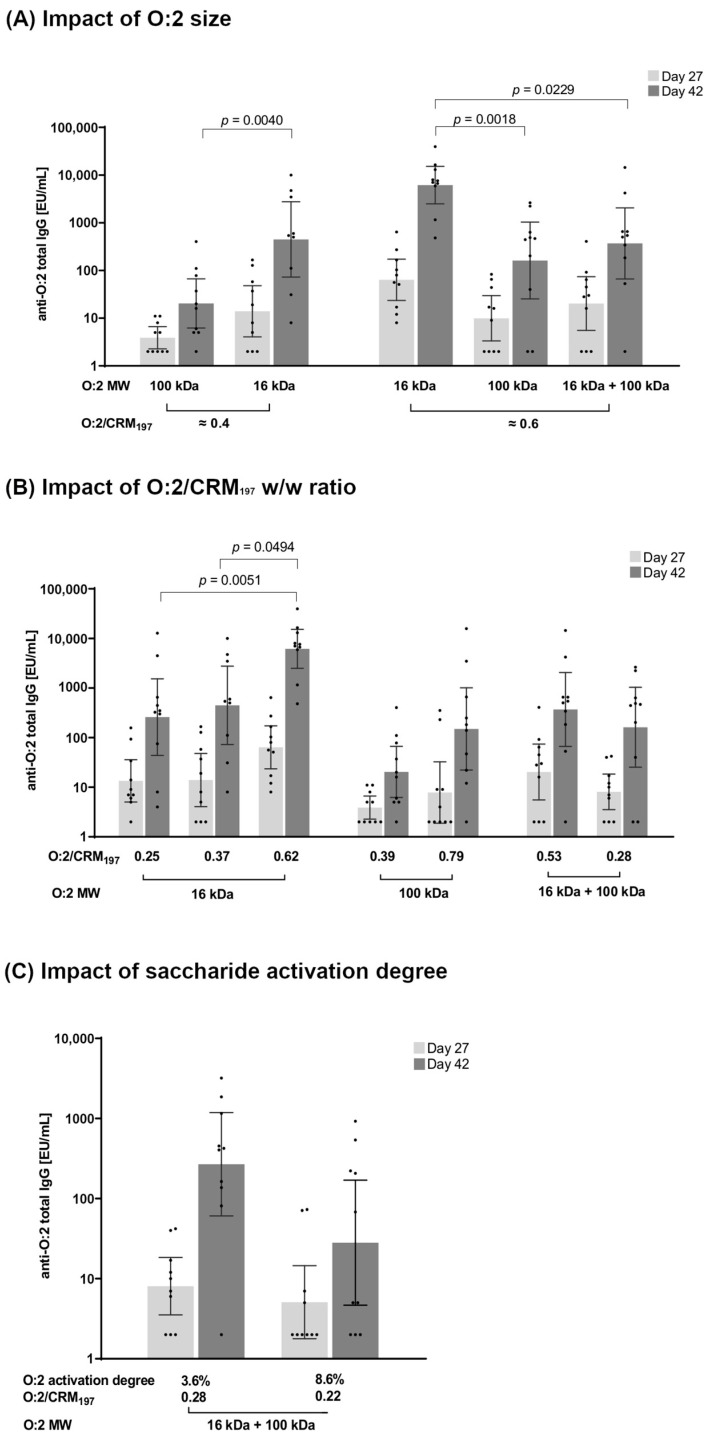
Summary graphs of anti-O:2 IgG geometric mean units (bars) and individual antibody levels (dots) are reported. (**A**) Impact on the immune response in mice of *S*. Paratyphi A O:2 size: O:2–CRM_197_ conjugates synthesized using O:2 with different sizes (16 kDa, 100 kDa, and 16 kDa + 100 kDa) and with a similar O:2/CRM_197_ *w*/*w* ratio were compared. (**B**) Impact of O:2/CRM_197_ *w*/*w* ratio: O:2–CRM_197_ conjugates synthesized from O:2 of a certain size but showing a different O:2/CRM_197_ *w*/*w* ratio were compared. (**C**) Impact of O:2 activation degree: O:2–CRM_197_ conjugates synthesized from O:2_[16kDa+100kDa]_ with a different activation degree were compared.

In general, all tested conjugates (Table 2), regardless of their specific structural differences, were immunogenic, with a significant booster effect after the second immunization (Figure 2).

#### 3.2.1. Effect of O:2 Size

O:2–CRM_197_ conjugates with a similar O:2/CRM_197_ *w*/*w* ratio but differing in O:2 MW (16 kDa, 100 kDa, or 100 kDa + 16 kDa) were compared. No significant differences were found among the conjugates after one injection. Two weeks after the second injection, O:2_[16kDa]_-CRM_197_ induced a significantly higher anti-O:2 IgG response than the analogous conjugate synthesized with O:2_[100kDa]_ (*p* = 0.0040). Both conjugates presented a similar O:2/CRM_197_ *w*/*w* ratio of 0.37 (O:2_[16kDa]_-CRM_197_) and 0.39 (O:2_[100kDa]_-CRM_197_) (Figure 2A). The same was found for conjugates at a similarly higher O:2/CRM_197_ *w*/*w* ratio (0.62 for O:2_[16kDa]_-CRM_197_, 0.65 for O:2_[100kDa]_-CRM_197_, and 0.53 for O:2_[16kDa+100kDa]_-CRM_197_), with O:2_[16kDa]_-CRM_197_ inducing the highest anti-O:2 IgG response (Figure 2A). The bactericidal activity of the antibodies induced by the conjugates was tested (Figure S5A), but many mice did not respond, and no significant differences were identified among groups.

#### 3.2.2. Effect of O:2/CRM_197_ Ratio

The impact of the O:2 loading on CRM_197_ was also investigated. Starting from O:2_[16kDa]_, O:2_[100kDa]_, and O:2_[16kDa+100kDa]_ populations, by working with different reaction conditions, it was possible to synthesize conjugates with different O:2/CRM_197_ *w*/*w* ratios (Table 2). O:2_[100kDa]_ conjugates 4 and 5 (Table 2) with an O:2/CRM_197_ *w*/*w* ratio of 0.39 and 0.79, respectively, elicited similar anti-O:2 IgG responses (Figure 2B) and serum bactericidal titers (Figure S5B). Also, conjugates 7 and 8 (Table 2) obtained from the O:2_[16kDa+100kDa]_ mixed population with an O:2/CRM_197_ *w*/*w* ratio of 0.28 and 0.53, respectively, induced similar responses. In contrast, for O:2_[16kDa]_ conjugate 3 with an O:2/CRM_197_ *w*/*w* ratio of 0.62, it induced significantly higher total anti-O:2 IgG response than the conjugates 1 and 2 (Table 2), with lower ratios of 0.25 (*p* = 0.0051) and 0.37 (*p* = 0.0494), respectively (Figure 2B).

#### 3.2.3. Effect of O:2 Activation Degree

To verify if different levels of saccharide activation with CDAP could impact the immunogenicity, we compared conjugates synthesized with the O:2_[16kDa+100kDa]_ mixed population, activated with a different percentage of nmol of cyanoester groups per nmol of sugar rings (e.g., the activation degree). Working with a CDAP/O:2 *w*/*w* ratio of 0.2 or 0.5, the degree of saccharide activation increased from 3.6% to 8.6%. The resulting conjugates (conjugates 8 and 9, Table 2) showed a similar O:2/CRM_197_ *w*/*w* ratio of 0.28 and 0.22 and were therefore characterized by different cross-linking. Once tested in mice, no significant difference in anti-O:2 IgG response was observed (Figure 2C), but the conjugate with a higher O:2 activation degree induced lower serum bactericidal titers than the conjugate with a lower activation (*p* = 0.0146, Figure S5C).

#### 3.2.4. Effect of O-Acetylation Levels

**Figure 3:**
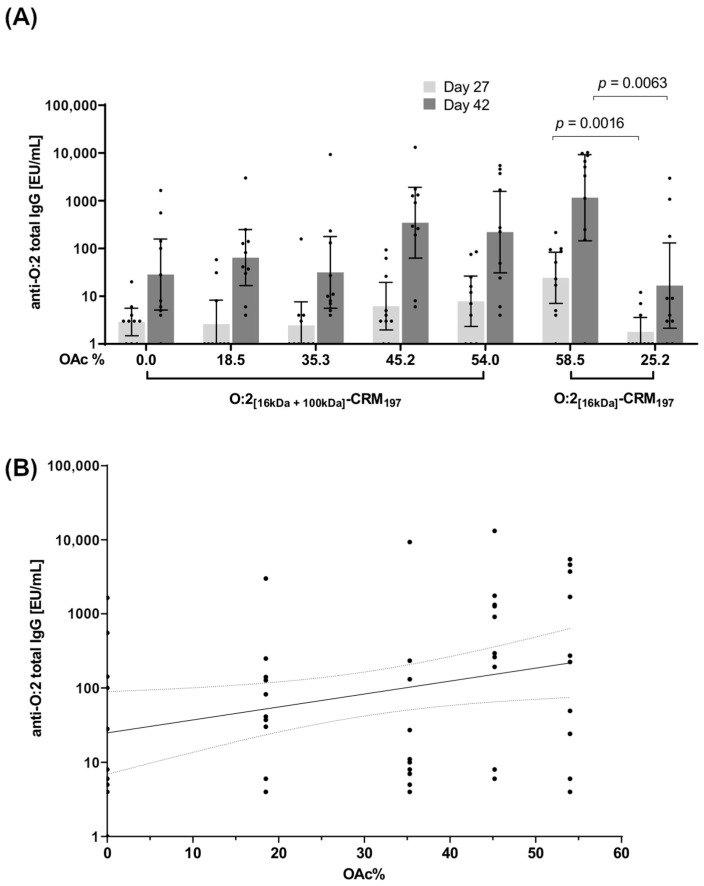
(**A**) Impact of O-acetylation level on anti-O:2 IgG response elicited in mice by O:2–CRM_197_ conjugates. Five different levels of O-acetylation were compared for O:2 _[16kDa+100kDa]_ and two different levels for O:2_[16kDa]_. Summary graphs of anti-O:2 IgG geometric mean units (bars with 95% CI) and individual antibody levels (dots) are reported. (**B**) Linear regression analysis for Log EU (Day 42) vs. OAc % with 95% CI.

To investigate the impact of O-acetylation on immunogenicity, the conjugates reported in Table 4 were tested in mice. The mice received two IP immunizations, with a 28-day interval, of 2.5 µg of conjugates (O:2 dose). In the case of O:2_[16kDa+100kDa]_, conjugates at five different O-acetylation levels (from 0% to 54%) were tested. The results were analyzed by regression analysis of ELISA results (log transformed) vs. OAc degree. All conjugates were immunogenic, with a booster response after the second immunization (Figure 3A). The analysis at day 42 (Figure 3B and Figure S6) showed a significant slope (*p* = 0.027) in the O-acetylation range 0–54%, with a non-significant lack of fit (*p* = 0.303), indicating that anti-O:2 IgG antibodies increase with increasing O-acetylation levels. We calculated a significant trend, with a total fold increase of 8.8 (CI_95%_ 1.3–59) EU/mL Geomean ratio, from 0% to 54% OAc levels. At day 27, the regression analysis did not evidence any significant trend in terms of slope (Figure S6).

The results were confirmed by testing O:2_[16kDa]_ conjugates at two different O-acetylation levels (58.5% and 25.2%, conjugates 6 and 7, respectively, Table 4); both at day 27 (*p* = 0.0016, Mann Whitney test) and at day 42 (*p* = 0.0063, Mann Whitney test), the conjugate with 58.5% O-acetylation elicited a higher response than the conjugate at 25.2% OAc (Figure 3A).

Functionality of the sera (day 42) was tested by serum bactericidal activity (SBA), but no differences nor trends were identified among the groups; the response was highly variable with a large number of no-responder mice.

### 3.3. Immunogenicity of O:2–CRM_197_ Conjugates in Rabbits

**Figure 4:**
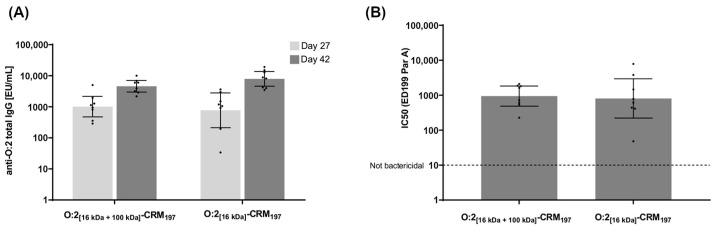
Immune response induced in rabbits by O:2–CRM_197_ conjugates differing in O:2 size. (**A**) Summary graphs of anti-O:2 IgG geometric mean units (bars with 95% CI) and individual antibody levels (dots) are reported. (**B**) Summary graphs of SBA titers as geometric mean units (bars with 95% CI) and individual SBA titers (dots) are reported.

Two conjugates synthesized from O:2_[16kDa+100kDa]_ and O:2_[16kDa]_ with the highest level of O-acetylation (and characterized by a similar OAg to protein ratio) were also tested in rabbits. This allowed us to investigate the immune response in a different animal species using a different immunization route (intramuscular versus intraperitoneal (IM vs. IP)). Both conjugates induced a sustained anti-O:2 IgG response 27 days after the first injection, which was boosted two weeks after the second injection (Figure 4A). The sera elicited by both conjugates were bactericidal (Figure 4B). No significant differences were observed between the two conjugates, neither in terms of anti-O:2 IgG nor in bactericidal activity.

### 3.4. Modeling of the O-Acetylation Stability

Since the O-acetylation degree was found to be a CQA for the O:2–CRM_197_ conjugate, we decided to also evaluate its stability with time by storing the conjugate at a pH of 6.5 and 7.4. An appropriate accelerated stability plan was designed. This information can be critical to ensuring the quality of the O:2–CRM_197_ conjugate over time [22].

**Figure 5:**
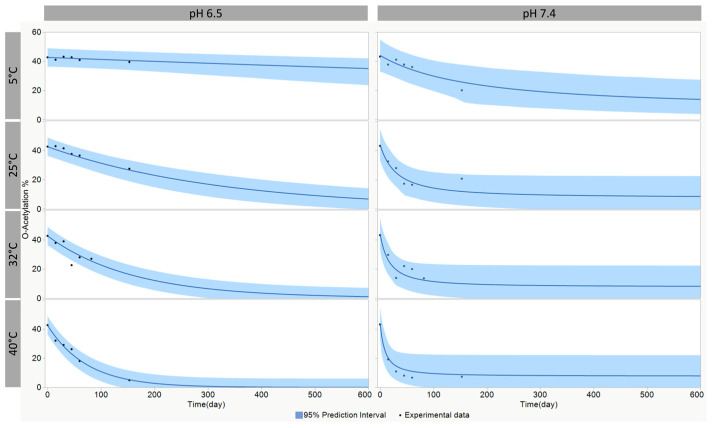
O-acetylation models obtained from the Šesták–Berggren truncated equation at different temperatures, with 95% prediction intervals for pH 6.5 and 7.4.

Considering that a potential decrease in O-acetylation would be slow and difficult to track at 2–8 °C in a short timeframe, we assessed the O-acetylation stability at higher temperatures to accelerate the degradation processes. The stability profile of the O:2–CRM_197_ conjugate was evaluated through a modern modeling approach, utilizing the truncated Šesták–Berggren equation (Table 3) for the statistical analysis of stability data at different incubation temperatures [34]. The stability data obtained at four different temperatures led to the identification of models that best describe the change in the OAc attribute as a function of time and temperature (Figure 5). A first-order kinetic model was identified at pH 6.5, and a reaction of order 1.7 was found for the conjugate stored at pH 7.4. The prediction in the real-time condition showed that, as expected, O-acetylation decreased faster at an increased pH; for example, at 5 °C, 25% residual O-acetylation is reached by the lower bound of the prediction interval in 54 days at pH 7.4 and in 530 days at pH 6.5.

## 4. Discussion

*Salmonella enterica* serovars Typhi and Paratyphi A are the causative agents of enteric fever in humans, accounting for around 13 million cases per year globally [35]. Although vaccines for typhoid fever have been developed and are in use, there are no vaccines for paratyphoid fever. Some *S.* Paratyphi A vaccines are in development based on either live-attenuated strains or the O-antigen (O:2) portion of lipopolysaccharide [36]. To develop a bivalent vaccine against both Typhi and Paratyphi A, we studied the design of the most suitable O:2–CRM_197_ conjugate.

We selected CRM_197_ as the carrier protein in alignment with our recently developed TCV, and we investigated the impact that other conjugate characteristics, such as saccharide MW [37], saccharide to protein ratio, saccharide activation degree, and O-acetylation level [38], can have on immunogenicity [39]. All these parameters can impact the magnitude and quality of the immune response and need to be monitored to ensure the consistency and efficacy of the glycoconjugate vaccine.

A random approach was selected to generate the conjugates, which considerably simplifies the manufacturing process compared to a selective approach; CDAP activation of the polysaccharide does not require purification of the activated O:2 before conjugation and does not need additional derivatization steps of either the polysaccharide or the protein with specific linkers.

We then investigated the impact of the O:2 polysaccharide size, as there are many examples of glycoconjugates for which this parameter can significantly impact immunogenicity [37]. For example, we found that the length of the Vi polysaccharide in Vi–CRM_197_ was critical to inducing a T-dependent response needed to protect infants [7]. In the case of O:2–CRM_197_, we observed that conjugates containing O:2 at a low MW (16 kDa vs. 100 kDa) induced stronger immunogenicity in mice. This was of particular relevance because it contradicts what was previously reported by Konadu et al. [16], who observed that an *S*. Paratyphi A O:2–TT conjugate with a low O:2 MW (23.4 kDa) induced significantly lower immunogenicity in mice than similar conjugates containing O:2 with a high MW (122 kDa) or with a mixed population of high and low MW (122 kDa + 23.4 kDa). Konadu et al. used EDAC chemistry to link O:2 to TT protein after polysaccharide derivatization with an ADH linker. The difference in the carrier protein and the chemistry used may result in cross-linked structures of different sizes and with different exposure and presentation to the immune system of the OAg chains, which may account for the different results. Also, in the case of *Salmonella* Typhimurium OAg [40,41], different results have been obtained in studies investigating the impact of sugar length on immunogenicity. In addition, it is well known that the immune response elicited by a glycoconjugate is influenced by the combination of multiple parameters [39].

In this study, we also verified that the O:2 to protein ratio had an impact on immunogenicity, specifically when a lower MW OAg was used to generate glycoconjugates.

Bacterial polysaccharides often contain substituents, such as OAc ester groups, which may constitute important immunodominant epitopes and can influence the immune response. It has been shown that OAc groups play a major role in the functional immune response elicited by some vaccines, such as *Neisseria meningitidis* serogroup A and *S.* Typhi Vi, while they do not seem to be important for others, such as meningococcal serogroups C, W, Y, and type III Group B Streptococcus [38]. In agreement with Konadu et al., who showed that O-acetylation of O:2–TT conjugates was essential to induce a functional humoral response in mice, we were able to demonstrate a trend of increasing anti-O:2 IgG antibodies with increasing O-acetylation levels, thus confirming the importance of monitoring this parameter.

Once critical attributes are identified, it is essential to verify their batch-to-batch consistency and stability over time. When a stability evaluation is exclusively based on real-time data, it may constitute an impairment for rapid vaccine development. Therefore, accelerated stability studies and predictive modeling represent key tools to predict stability in a much shorter timeframe. Such an approach has been used here to predict O-acetylation stability in two different storage conditions, and it informed us of the optimal pH needed to preserve it.

When we compared the immunogenicity results obtained in mice with what was found in rabbits, we noticed some differences; in general, mice generated lower bactericidal activity and less homogeneous anti-O:2 IgG responses compared to rabbits, and some conjugate parameters, such as O:2 MW, had a different impact on immunogenicity. Despite not knowing how preclinical results will translate in humans and which animal model can be more predictive, animal studies can still represent a starting point to guide the design of a vaccine by selecting the candidates with the highest probability of eliciting an appropriate immune response in clinical studies.

## 5. Conclusions

In conclusion, the preclinical studies we described here guided us to select an *S*. Paratyphi A conjugate with an O:2 molecular size around 16 KDa, an O:2/CRM_197_ ratio > 0.37, and OAc levels as close as possible to the native O-antigen. It would be beneficial to compare some of the conjugates differing in the critical quality parameters identified through this work in an in vivo model to confirm their impact on the ability to protect [42,43,44]. We have combined the O:2–CRM_197_ conjugate with a TCV in a bivalent formulation against enteric fever, and a phase 1 clinical study to test its safety and immunogenicity is currently ongoing (NCT05613205).

## Figures and Tables

**Table 1:** Analytical characterization of the O:2 populations used for conjugation with CRM_197_.

O:2	Source	MW (M_p_)	Glucosylation %	O-acetylation %
O:2_[16kDa+100kDa]_	ED199 ΔtolR strain	100 kDa + 16 kDa (mix)	79.0	60.0
O:2_[100kDa]_ O:2_[16kDa]_	ED199 ΔtolR strain (populations isolated by SEC)	only 100 kDa only 16 kDa	83.2 85.2	47.8 44.0

**Table 2:** O:2–CRM_197_ conjugates differing by O:2 size, O:2/CRM_197_ *w*/*w* ratio, and O:2 activation degree. Conjugates were synthesized using O:2 populations of different sizes and changing the weight proportion of O:2, CRM_197_, and CDAP in reaction.

Nr	Conjugate	O:2 Size (kDa)	Conjugation Conditions (Weight Proportion)			O:2 Activation *	Conjugate
			PS	CRM_197_	CDAP	%	O:2/CRM_197_ *w*/*w* Ratio **
1	O:2_[16kDa]_–CRM_197_	16	1	2	0.2	nd	0.25
2	O:2_[16kDa]_–CRM_197_	16	1	0.5	0.2	nd	0.37
3	O:2_[16kDa]_–CRM_197_	16	1	0.5	0.5	nd	0.62
4	O:2_[100kDa]_–CRM_197_	100	1	2	0.2	nd	0.39
5	O:2_[100kDa]_–CRM_197_	100	1	0.5	0.2	nd	0.79
6	O:2_[100kDa]_–CRM_197_	100	1	0.5	0.5	nd	0.65
7	O:2_[16kDa+100kDa]_–CRM_197_	16 + 100	1	0.5	0.2	nd	0.53
8	O:2_[16kDa+100kDa]_–CRM_197_	16 + 100	1	2	0.2	3.6	0.28
9	O:2_[16kDa+100kDa]_–CRM_197_	16 + 100	1	2	0.5	8.6	0.22

* Expressed as nmol of activated groups per nmol of sugar rings. ** Expressed as *w*/*w* ratio between O:2 and conjugated CRM_197_. nd: not determined.

**Table 3:** Parameters of the Šesták–Berggren truncated equation found by the stability modeling.

$\frac{d\alpha }{dt}=A{exp}^{-\frac{E}{RT}}{\left(1-\alpha \right)}^{n}$	A (1/s)	E (J/mol)	n	Y_initial_ (T0)	Y_end_ (T∞)
O-acetylation pH 6.5	1.176 × 10^6^	77,149.5	1	42.7	2.2 × 10^−5^
O-acetylation pH 7.4	9330	59,286.1	1.7	44.0	7.7

A: pre-exponential factor; E: activation energy; n: reaction order; Y_initial_: parameter (O-acetylation) value at starting time; and Y_end_: parameter (O-acetylation) value at asymptote of the curve.

**Table 4:** Analytical characterization of O:2–CRM_197_ conjugates synthesized through CDAP chemistry starting from O:2_[16kDa+100kDa]_ and O:2_[16kDa]_ at different O-acetylation levels.

Nr	O:2 Size (kDa)	Conjugate	Conjugate O:2/CRM_197_ *w*/*w* Ratio	OAc Level %	Free O:2 %
1	16 kDa + 100 kDa	O:2_[54% OAc]_–CRM_197_	0.69	54	4.7
2	16 kDa + 100 kDa	O:2_[45.2% OAc]_–CRM_197_	0.49	45.2	2.8
3	16 kDa + 100 kDa	O:2_[35.3% OAc]_–CRM_197_	0.42	35.3	3.6
4	16 kDa + 100 kDa	O:2_[18.5% OAc]_–CRM_197_	0.41	18.5	2.9
5	16 kDa + 100 kDa	O:2_[0% OAc]_–CRM_197_	0.38	0	2.5
6	16 kDa	O:2_[58.5% OAc]_–CRM_197_	0.39	58.5	6.1
7	16 kDa	O:2_[25.2% OAc]_–CRM_197_	0.36	25.2	5.6

## Data Availability

The authors declare that the data are contained within the article and in the Supplementary Materials.

## Supplementary Materials

The following supporting information can be downloaded at: https://www.mdpi.com/article/10.3390/vaccines12111272/s1, Table S1: O:2_[16kDa+100kDa]_ O-acetylation % after treatment with an increasing concentration of ammonia; Figure S1: HPLC-SEC of O:2_[16kDa+100kDa]_, O:2_[16kDa]_, and O:2_[100kDa]_; Figure S2: Mathematical function, with a 95% prediction interval, correlating the residual O:2 OAc level to the ammonia concentration; Figure S3: Regression analysis of OAc vs. ammonia; Figure S4: HPLC-SEC profiles of O:2–CRM_197_ conjugates at different levels of O-acetylation; Figure S5: SBA titers (mice); Figure S6: Regression for the log(ELISA unit) of O:2_[16kDa+100kDa]_–CRM_197_ conjugates in mice.

## References

[1] (2022). Global burden of bacterial antimicrobial resistance in 2019: A systematic analysis. Lancet.

[2] Sallam M., Snygg J., Allam D., Kassem R. (2024). From Protection to Prevention: Redefining Vaccines in the Context of Antimicrobial Resistance. Cureus.

[3] Hasso-Agopsowicz M., Sparrow E., Cameron A.M., Sati H., Srikantiah P., Gottlieb S., Bentsi-Enchill A., Le Doare K., Hamel M., Giersing B.K. (2024). The role of vaccines in reducing antimicrobial resistance: A review of potential impact of vaccines on AMR and insights across 16 vaccines and pathogens. Vaccine.

[4] Zavaleta-Monestel E., Hasselmyr Hasselmyr S., García-Montero J., Arguedas-Chacón S., Rojas-Chinchilla C., Díaz-Madriz J.P. (2024). The Impact of Vaccination as a Strategy to Combat Bacterial Antimicrobial Resistance. Cureus.

[5] Patel P.D., Liang Y., Meiring J.E., Chasweka N., Patel P., Misiri T., Mwakiseghile F., Wachepa R., Banda H.C., Shumba F. (2024). Efficacy of typhoid conjugate vaccine: Final analysis of a 4-year, phase 3, randomised controlled trial in Malawian children. Lancet.

[6] World Health Organization (2019). Typhoid vaccines: WHO position paper, March 2018—Recommendations. Vaccine.

[7] Micoli F., Bjarnarson S.P., Arcuri M., Aradottir Pind A.A., Magnusdottir G.J., Necchi F., Di Benedetto R., Carducci M., Schiavo F., Giannelli C. (2020). Short Vi-polysaccharide abrogates T-independent immune response and hyporesponsiveness elicited by long Vi-CRM(197) conjugate vaccine. Proc. Natl. Acad. Sci. USA.

[8] Rondini S., Micoli F., Lanzilao L., Hale C., Saul A.J., Martin L.B. (2011). Evaluation of the immunogenicity and biological activity of the Citrobacter freundii Vi-CRM197 conjugate as a vaccine for Salmonella enterica serovar Typhi. Clin. Vaccine Immunol..

[9] Arcuri M., Di Benedetto R., Cunningham A.F., Saul A., MacLennan C.A., Micoli F. (2017). The influence of conjugation variables on the design and immunogenicity of a glycoconjugate vaccine against Salmonella Typhi. PLoS ONE.

[10] Rahman S.I.A., Nguyen T.N.T., Khanam F., Thomson N.R., Dyson Z.A., Taylor-Brown A., Chowdhury E.K., Dougan G., Baker S., Qadri F. (2021). Genetic diversity of Salmonella Paratyphi A isolated from enteric fever patients in Bangladesh from 2008 to 2018. PLoS Negl. Trop. Dis..

[11] (2019). The global burden of typhoid and paratyphoid fevers: A systematic analysis for the Global Burden of Disease Study 2017. Lancet Infect. Dis..

[12] Zhou Z., McCann A., Weill F.X., Blin C., Nair S., Wain J., Dougan G., Achtman M. (2014). Transient Darwinian selection in Salmonella enterica serovar Paratyphi A during 450 years of global spread of enteric fever. Proc. Natl. Acad. Sci. USA.

[13] Zellweger R.M., Basnyat B., Shrestha P., Prajapati K.G., Dongol S., Sharma P.K., Koirala S., Darton T.C., Dolecek C., Thompson C.N. (2017). A 23-year retrospective investigation of Salmonella Typhi and Salmonella Paratyphi isolated in a tertiary Kathmandu hospital. PLoS Negl. Trop. Dis..

[14] Irfan S., Hasan Z., Qamar F.N., Ghanchi N., Ashraf J., Kanji A., Razzak S.A., Greig D., Nair S., Hasan R. (2023). Correction: Ceftriaxone resistant Salmonella enterica serovar Paratyphi A identified in a case of enteric fever: First case report from Pakistan. BMC Infect. Dis..

[15] Micoli F., Rondini S., Gavini M., Lanzilao L., Medaglini D., Saul A., Martin L.B. (2012). O:2-CRM(197) conjugates against Salmonella Paratyphi A. PLoS ONE.

[16] Konadu E., Shiloach J., Bryla D.A., Robbins J.B., Szu S.C. (1996). Synthesis, characterization, and immunological properties in mice of conjugates composed of detoxified lipopolysaccharide of Salmonella paratyphi A bound to tetanus toxoid with emphasis on the role of O acetyls. Infect. Immun..

[17] Konadu E.Y., Lin F.Y., Hó V.A., Thuy N.T., Van Bay P., Thanh T.C., Khiem H.B., Trach D.D., Karpas A.B., Li J. (2000). Phase 1 and phase 2 studies of Salmonella enterica serovar paratyphi A O-specific polysaccharide-tetanus toxoid conjugates in adults, teenagers, and 2- to 4-year-old children in Vietnam. Infect. Immun..

[18] MacLennan C.A., Stanaway J., Grow S., Vannice K., Steele A.D. (2023). Salmonella Combination Vaccines: Moving Beyond Typhoid. Open Forum. Infect. Dis..

[19] Chettri D., Chirania M., Boro D., Verma A.K. (2024). Glycoconjugates: Advances in modern medicines and human health. Life Sci..

[20] Costantino P., Rappuoli R., Berti F. (2011). The design of semi-synthetic and synthetic glycoconjugate vaccines. Expert Opin. Drug Discov..

[21] Berti F., Adamo R. (2018). Antimicrobial glycoconjugate vaccines: An overview of classic and modern approaches for protein modification. Chem. Soc. Rev..

[22] Ravenscroft N., Cescutti P., Gavini M., Stefanetti G., MacLennan C.A., Martin L.B., Micoli F. (2015). Structural analysis of the O-acetylated O-polysaccharide isolated from Salmonella paratyphi A and used for vaccine preparation. Carbohydr. Res..

[23] Nappini R., Alfini R., Durante S., Salvini L., Raso M.M., Palmieri E., Di Benedetto R., Carducci M., Rossi O., Cescutti P. (2024). Modeling 1-Cyano-4-dimethylaminopyridine Tetrafluoroborate (CDAP) Chemistry to Design Glycoconjugate Vaccines with Desired Structural and Immunological Characteristics. Vaccines.

[24] van der Put R.M.F., Metz B., Pieters R.J. (2023). Carriers and Antigens: New Developments in Glycoconjugate Vaccines. Vaccines.

[25] Micoli F., Giannelli C., Di Benedetto R. (2021). O-Antigen Extraction, Purification, and Chemical Conjugation to a Carrier Protein. Methods Mol. Biol..

[26] Raso M.M., Vassallo O., Micoli F., Giannelli C. (2021). Comparison and Optimization of Quantification Methods for Shigella flexneri Serotype 6 O-antigen Containing Galacturonic Acid and Methyl-Pentose. Int. J. Mol. Sci..

[27] Hitri K., Kuttel M.M., De Benedetto G., Lockyer K., Gao F., Hansal P., Rudd T.R., Beamish E., Rijpkema S., Ravenscroft N. (2019). O-acetylation of typhoid capsular polysaccharide confers polysaccharide rigidity and immunodominance by masking additional epitopes. Vaccine.

[28] Stefanetti G., Rondini S., Lanzilao L., Saul A., MacLennan C.A., Micoli F. (2014). Impact of conjugation chemistry on the immunogenicity of S. Typhimurium conjugate vaccines. Vaccine.

[29] Micoli F., Alfini R., Giannelli C. (2022). Methods for Assessment of OMV/GMMA Quality and Stability. Methods Mol. Biol..

[30] Hestrin S. (1949). The reaction of acetylcholine and other carboxylic acid derivatives with hydroxylamine, and its analytical application. J. Biol. Chem..

[31] Gasperini G., Massai L., De Simone D., Raso M.M., Palmieri E., Alfini R., Rossi O., Ravenscroft N., Kuttel M.M., Micoli F. (2024). O-Antigen decorations in Salmonella enterica play a key role in eliciting functional immune responses against heterologous serovars in animal models. Front. Cell. Infect. Microbiol..

[32] Necchi F., Saul A., Rondini S. (2018). Setup of luminescence-based serum bactericidal assay against Salmonella Paratyphi A. J. Immunol. Methods.

[33] Mylona E., Sanchez-Garrido J., Hoang Thu T.N., Dongol S., Karkey A., Baker S., Shenoy A.R., Frankel G. (2021). Very long O-antigen chains of Salmonella Paratyphi A inhibit inflammasome activation and pyroptotic cell death. Cell Microbiol..

[34] Campa C., Pronce T., Paludi M., Weusten J., Conway L., Savery J., Richards C., Clénet D. (2021). Use of Stability Modeling to Support Accelerated Vaccine Development and Supply. Vaccines.

[35] Global Burden of Disease Collaborative Network (2020). Global Burden of Disease Study 2019.

[36] Baliban S.M., Lu Y.J., Malley R. (2020). Overview of the Nontyphoidal and Paratyphoidal Salmonella Vaccine Pipeline: Current Status and Future Prospects. Clin. Infect. Dis..

[37] Stefanetti G., MacLennan C.A., Micoli F. (2022). Impact and Control of Sugar Size in Glycoconjugate Vaccines. Molecules.

[38] Berti F., De Ricco R., Rappuoli R. (2018). Role of O-Acetylation in the Immunogenicity of Bacterial Polysaccharide Vaccines. Molecules.

[39] Micoli F., Stefanetti G., MacLennan C.A. (2023). Exploring the variables influencing the immune response of traditional and innovative glycoconjugate vaccines. Front. Mol. Biosci..

[40] Svenson S.B., Lindberg A.A. (1981). Artificial Salmonella vaccines: Salmonella typhimurium O-antigen-specific oligosaccharide-protein conjugates elicit protective antibodies in rabbits and mice. Infect. Immun..

[41] Rondini S., Micoli F., Lanzilao L., Gavini M., Alfini R., Brandt C., Clare S., Mastroeni P., Saul A., MacLennan C.A. (2015). Design of glycoconjugate vaccines against invasive African Salmonella enterica serovar Typhimurium. Infect. Immun..

[42] Zhu C., Xiong K., Chen Z., Hu X., Li J., Wang Y., Rao X., Cong Y. (2015). Construction of an attenuated Salmonella enterica serovar Paratyphi A vaccine strain harboring defined mutations in htrA and yncD. Microbiol. Immunol..

[43] Roland K.L., Tinge S.A., Kochi S.K., Thomas L.J., Killeen K.P. (2010). Reactogenicity and immunogenicity of live attenuated Salmonella enterica serovar Paratyphi A enteric fever vaccine candidates. Vaccine.

[44] Santander J., Curtiss R. (2010). Salmonella enterica Serovars Typhi and Paratyphi A are avirulent in newborn and infant mice even when expressing virulence plasmid genes of Salmonella Typhimurium. J. Infect. Dev. Ctries.

