# Supplementary material for: Design of a Glycoconjugate Vaccine Against Salmonella Paratyphi A
Source: Vaccines (Basel). 2024 Nov 12;12(11):1272. doi: 10.3390/vaccines12111272 (PMC11599127; doi:10.3390/vaccines12111272)
Supplement: Supplementary file 1 [file vaccines-12-01272-s001.zip › vaccines-3254966-supplementary.pdf]

Supporting information

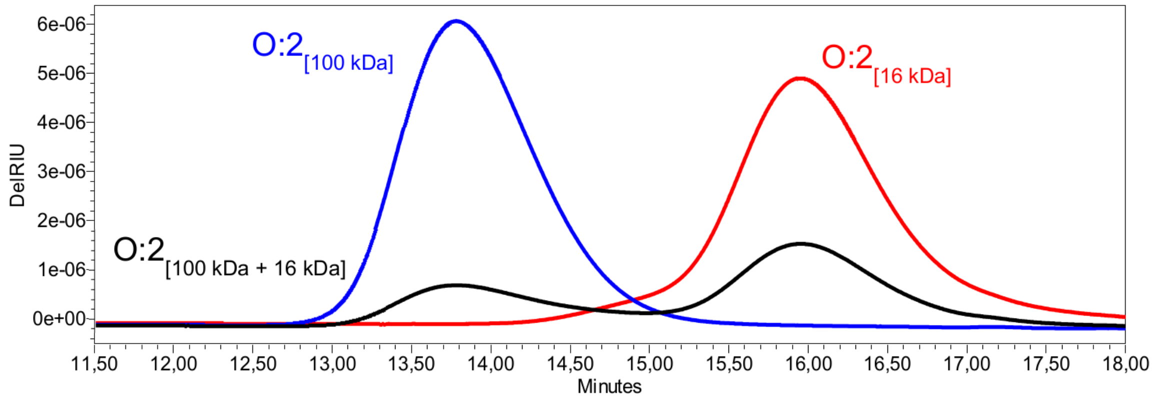

Figure S1. HPLC SEC of O:2$_{[16kDa + 100kDa]}$, O:2$_{[16kDa]}$, O:2$_{[100kDa]}$ (dRI profile), samples run on Tosoh TSK gel 3000 PW$_{XL}$ column

Table S1: O:2 $_{[16 kDa + 100 kDa]}$ O-acetylation % after treatment with increasing concentration of ammonia from 5 mM up to 1 M.

NH_4OH (mM)	O-Ac %
0	60
5	35.7
12.5	27.6
25	21
50	10.7
100	6.1
200	2.2
1000	0

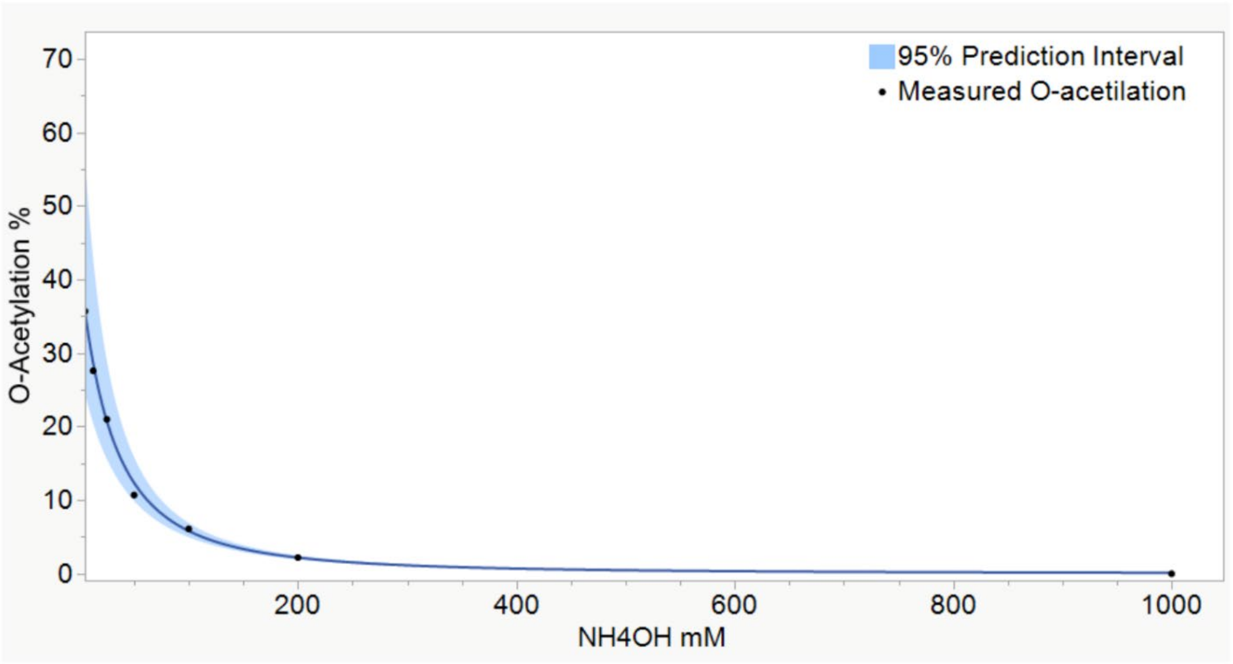

Figure S2. Treatment of O:2 with increasing concentration of ammonia allowed to obtain partially de-O-acetylated O:2. The graph reports the mathematical function, with 95% prediction interval, correlating the residual O:2 OAc level to ammonia concentration.

Regression Analysis: OAc% versus [NH4OH]

Method

Box-Cox transformation λ = -0.5

Analysis of Variance for Transformed Response

Source	DF	Adj SS	Adj MS	F-Value	P-Value
Regression	1	0.211784	0.211784	1458.26	0.000
[NH4OH]	1	0.211784	0.211784	1458.26	0.000
Error	5	0.000726	0.000145		
Total	6	0.212510			

Model Summary for Transformed Response

S	R-sq	R-sq(adj)	R-sq(pred)
0.0120512	99.66%	99.59%	99.49%

Coefficients for Transformed Response

Term	Coef	SE Coef	T-Value	P-Value	VIF
Constant	-0.15445	0.00594	-25.98	0.000	
[NH4OH]	-0.002601	0.000068	-38.19	0.000	1.00

Regression Equation

-OAc%^-0.5 = -0.15445 - 0.002601 [NH4OH]

Figure S3: Regression analysis OAc vs [Ammonia] (calculated with data in the range of 0-200 mM)

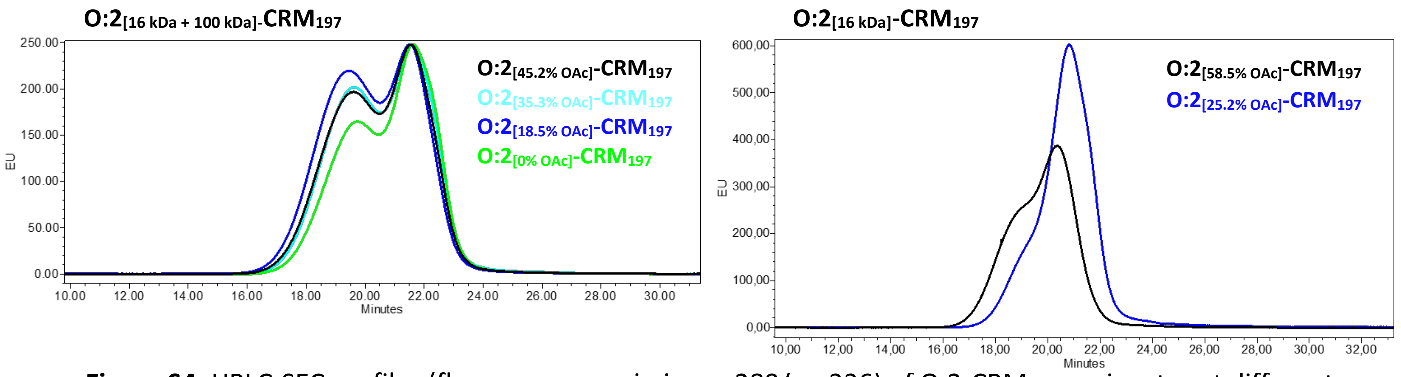

Figure S4: HPLC-SEC profiles (fluorescence emission ex280/em336) of O:2-CRM_{197} conjugates at different levels of O-acetylation. Samples run on Tosoh TSK gel 6000+5000 PW columns connected in series; eluent: 0.1 M NaH_2PO_4, 0.1 M NaCl, 5% ACN, pH 7.2; flow rate: 0.5 mL/min.

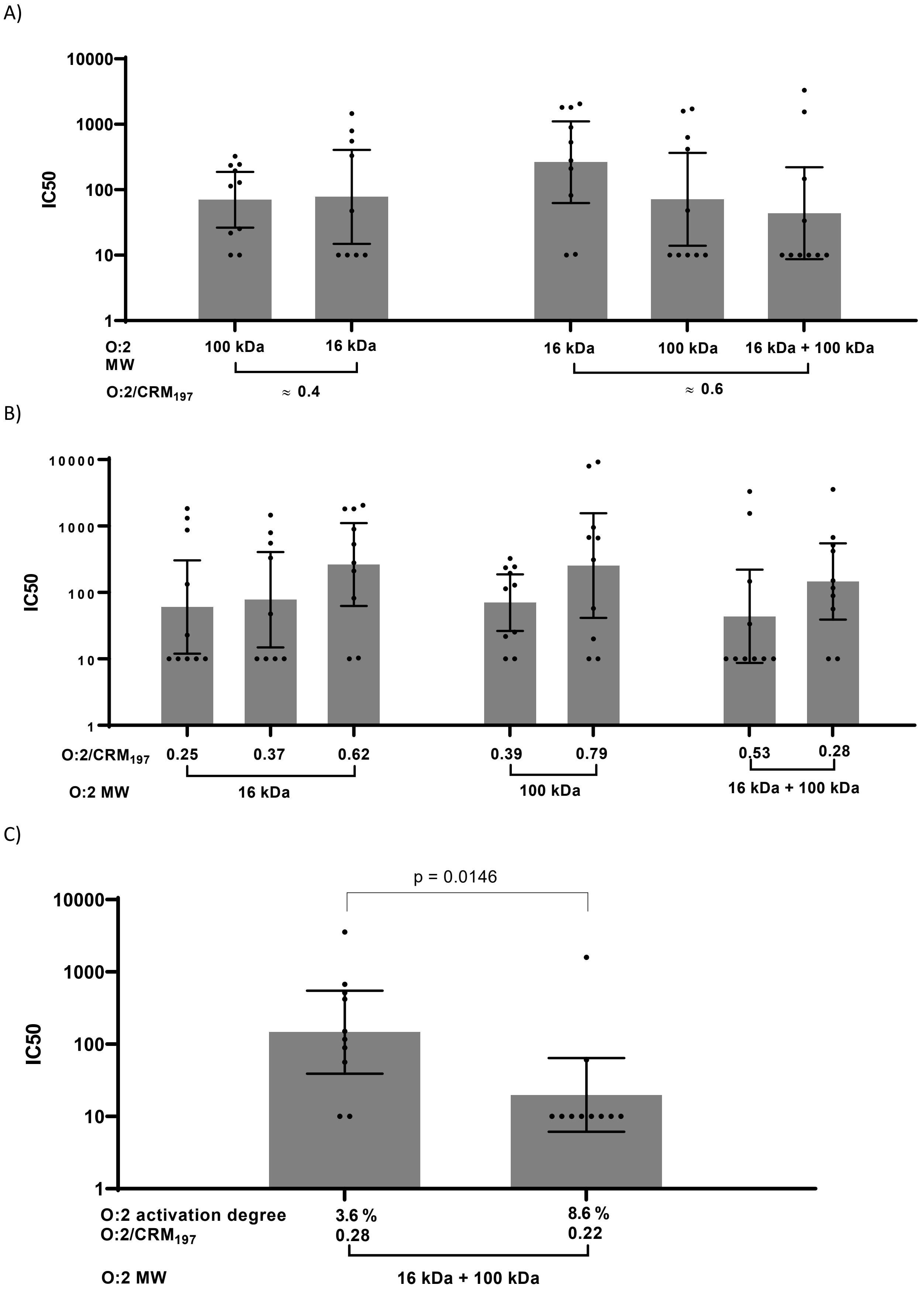

Figure S5: SBA titers (mice). A) impact of the O:2 size; B) O:2/CRM_{197} w/w ratio, C) cross-linking

Regression Analysis: log EU D27 versus OAc

Analysis of Variance

Source	DF	Seq SS	Contribution	Adj SS	Adj MS	F-Value	P-Value
Regression	1	1.2379	5.72%	1.2379	1.2379	2.91	0.094
OAc	1	1.2379	5.72%	1.2379	1.2379	2.91	0.094
Error	48	20.4091	94.28%	20.4091	0.4252		
Lack-of-Fit	3	0.9684	4.47%	0.9684	0.3228	0.75	0.530
Pure Error	45	19.4406	89.81%	19.4406	0.4320		
Total	49	21.6470	100.00%				

Model Summary

S	R-sq	R-sq(adj)	PRESS	R-sq(pred)
0.652065	5.72%	3.75%	21.9621	0.00%

Coefficients

Term	Coef	SE Coef	95% CI	T-Value	P-Value	VIF
Constant	0.340	0.173	(-0.007; 0.687)	1.97	0.055	
OAc	0.00815	0.00477	(-0.00145; 0.01774)	1.71	0.094	1.00

Regression Equation

log EU D27 = 0.340 + 0.00815 OAc

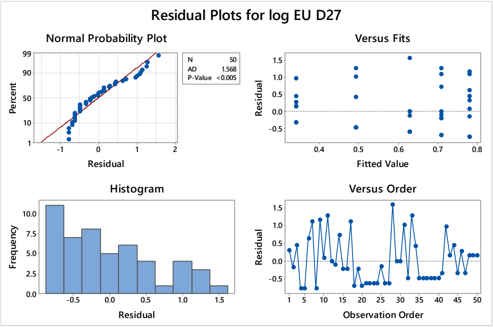

Regression Analysis: log EU D42 versus OAc

Analysis of Variance

Source	DF	Seq SS	Contribution	Adj SS	Adj MS	F-Value	P-Value
Regression	1	5.687	9.82%	5.687	5.687	5.23	0.027
OAc	1	5.687	9.82%	5.687	5.687	5.23	0.027
Error	48	52.208	90.18%	52.208	1.088		
Lack-of-Fit	3	4.014	6.93%	4.014	1.338	1.25	0.303
Pure Error	45	48.194	83.24%	48.194	1.071		
Total	49	57.895	100.00%				

Model Summary

S	R-sq	R-sq(adj)	PRESS	R-sq(pred)
1.04292	9.82%	7.94%	56.7294	2.01%

Coefficients

Term	Coef	SE Coef	95% CI	T-Value	P-Value	VIF
Constant	1.394	0.276	(0.838; 1.949)	5.04	0.000	
OAc	0.01746	0.00764	(0.00211; 0.03281)	2.29	0.027	1.00

Regression Equation

log EU D42 = 1.394 + 0.01746 OAc

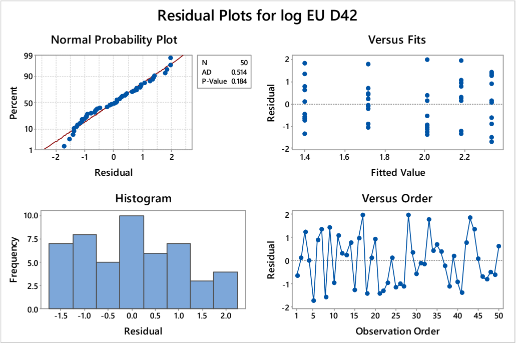

Figure S6: regression for log(ELISA unit) of O:2$_{[16\ kDa\ +\ 100\ kDa]}$-CRM$_{197}$ conjugates in mice study
